# Zinc Status Affects Glucose Homeostasis and Insulin Secretion in Patients with Thalassemia

**DOI:** 10.3390/nu7064296

**Published:** 2015-06-02

**Authors:** Ellen B. Fung, Ginny Gildengorin, Siddhant Talwar, Leah Hagar, Ashutosh Lal

**Affiliations:** 1 Department of Hematology/Oncology at the UCSF Benioff Children’s Hospital, 747 52nd Street Oakland, CA 94609, USA; E-Mail: alal@mail.cho.org; 2 Children’s Hospital Oakland Research Institute, 5700 Martin Luther King Jr Way, Oakland, CA 94609, USA; E-Mails: talwarsiddhant@hotmail.com (S.T.); indystar23@gmail.com (L.H.); 3 The Pediatric Clinical Research Center, 747 52nd Street Oakland, CA 94609, USA; E-Mail: ggildengorin@mail.cho.org

**Keywords:** Zinc, Diabetes, Thalassemia, Glucose intolerance

## Abstract

Up to 20% of adult patients with Thalassemia major (Thal) live with diabetes, while 30% may be zinc deficient. The objective of this study was to explore the relationship between zinc status, impaired glucose tolerance and insulin sensitivity in Thal patients. Charts from thirty subjects (16 male, 27.8 ± 9.1 years) with Thal were reviewed. Patients with low serum zinc had significantly lower fasting insulin, insulinogenic and oral disposition indexes (all *p* < 0.05) and elevated glucose response curve, following a standard 75 g oral load of glucose compared to those with normal serum zinc after controlling for baseline (group × time interaction *p* = 0.048). Longitudinal data in five patients with a decline in serum zinc over a two year follow up period (−19.0 ± 9.6 μg/dL), showed consistent increases in fasting glucose (3.6 ± 3.2 mg/dL) and insulin to glucose ratios at 120 min post glucose dose (*p* = 0.05). Taken together, these data suggest that the frequently present zinc deficiency in Thal patients is associated with decreased insulin secretion and reduced glucose disposal. Future zinc trials will require modeling of oral glucose tolerance test data and not simply measurement of static indices in order to understand the complexities of pancreatic function in the Thal patient.

## 1. Introduction

Thalassemia (Thal) is a heterogeneous group of autosomal recessive disorders of limited or absent production of the alpha or beta globin chain of hemoglobin; classified according to which globin chain is deficient. The disease is characterized by varying degrees of ineffective hematopoiesis, chronic anemia, intermittent hemolysis and iron overload [1]. Many patients require frequent blood transfusions to sustain life and prevent severe skeletal deformities [1]. Thal has an incidence near 60% in some regions of Southeast Asia, though estimates are much lower in North America, limited to less than 1000 patients in the U.S. and Canada combined [2,3]. 

Approximately 9% to 19% of adult patients with transfusion dependent β-Thal major suffer from diabetes [4,5]. Diabetes is clinically characterized by hyperglycemia due to either low circulating concentrations of, or decreased sensitivity to, insulin. Patients with Thal typically exhibit β-cell or insulin insufficiency, and may develop diabetes due to toxic levels of iron in their pancreas, one of the strongest predictors of β-cell destruction [6]. By contrast, hyperinsulinemia, secondary to insulin resistance, with normal glucose tolerance has also been observed [7]. It has been suggested that in this subset of patients with Thal, diabetes may be reversible, if identified early and total body iron is reduced [8]. However, diabetes is a cause of significant morbidity even in historically well-chelated patients with β thalassemia who have low tissue liver iron concentrations [4]. Clearly, the varied clinical presentations of diabetes in Thal raise the possibility of multiple contributing factors or etiologies.

In patients without thalassemia, there is a rich body of literature focused on the “diabetogenic effects” of altered zinc status [9]. Zinc supplementation has even been suggested as an adjunct therapy in the management of non-thalassemia related diabetes [10]. Recently, our group has shown that a functional zinc deficiency exists in a contemporary sample of healthy Thal patients [11]. An estimated 20% to 30% of patients with Thal are zinc deficient [11]. The high prevalence is thought to be related to a combination of increased urinary losses compounded by elevated requirements. The relationship between zinc status and diabetes has been suggested in Thal patients residing in Iran [12]. 

In this study, we sought to explore the relationship between zinc status, impaired glucose tolerance and insulin sensitivity in a contemporary sample of patients with Thal residing in the U.S. 

## 2. Materials and Methods 

The project presented herein was a retrospective chart review to explore the relationship between zinc status and response to a 75 g glucose challenge. The study was reviewed and considered exempt by the Institutional Review Board at the Children’s Hospital & Research Center, Oakland. It was conducted on all patients with a diagnosis of beta thalassemia major currently enrolled in our hematology clinic (*n* = 111). These subjects were selected for review as they typically receive an oral glucose tolerance test (OGTT) for diabetes screen. Abstracted variables included OGTT completed within the past eight years, and a fasting serum zinc sample drawn within eight months of the oral glucose tolerance test (OGTT). Basic clinical information regarding age, gender, thalassemia diagnosis and iron status was also obtained. 

Data were first plotted and normality tests run to check for outliers, ranges and distribution assumptions. Summary statistics were then computed including means, standard error of the mean (SEM), standard deviations (SD) and 95% confidence intervals (CI) for all the variables on each time point in each group. Low zinc was defined as serum concentration ≤71 μg/dL. We used a slightly more conservative cut-off for low serum zinc than IZinCG recommendations for males and females due to the young age of the patients, high percentage of hypogonadal females on replacement hormones, possibility of non-fasting samples, the likelihood for hemolysis in the sample [13,14]. Abnormal fasting glucose was defined as any value ≥100 mg/dL and abnormal 120 min blood glucose value post glucose load was defined as any value >140 mg/dL [15]. Impaired Fasting Glucose (IFG), was defined as a fasting glucose between 100 and 125 mg/dL and impaired Glucose Tolerance (IGT) was defined as a 2-h sample result from the oral glucose tolerance test between 140 and 199 mg/dL [15].

A number of markers of beta cell function and insulin sensitivity were calculated as described below as no one method was found robust enough to describe glucose and insulin metabolism in this population. Area under the curve (AUC) was calculated for glucose and insulin response to the oral glucose 75-g load. Whole body insulin sensitivity index homeostatic model (ISI_comp_): was calculated: 10,000sqrt[(FPGxFPI) × (MeanGlucose × MeanInsulin)] as previously described [16]. Homeostatic model assessment (HOMA-IR) (FPGxFPI/405) was used to quantify insulin resistance according to Matsuda [16]. Oral glucose Insulinogenic Index (IGI) was calculated: [Insulin30_min_ − Insulin0_min_]/[Glucose30_min_ − Glucose0_min_] according to Hanson as a marker of beta cell function [17]. Disposition index ODI [IGI × ISI] was used as an indicator of beta cell function, adjusted for insulin sensitivity (29). Raw insulin values were used in presentation, but log transformed before analysis due to skewness.

Categorical variables were analyzed by chi-square test, and continuous variables by *t*-test, with pooled (for equal variances) or Satterwaite (for unequal variances) *p*-value. Analysis of variance models with repeated measures over time (ANOVA) were run, to explore group × time interactions. If interactions were observed, the groups (normal and low zinc) were explored separately. ANOVA models with repeated measures were used to compare groups, while controlling for baseline measures. Multiple comparisons were adjusted using Tukey-Kramer’s method of adjustment. Statistical analyses were conducted using Stata 9.2 (Stata, Inc., College Station, TX, USA) and SAS version 9.3 (SAS Institute, 2010 Cary, NC, USA). A significance level of <0.05 was used for all statistical tests, with a p value of <0.1 considered a trend.

## 3. Results

Thirty patients with thalassemia (27.8 ± 9.1 years, range: 14–54 years, 16 male) had at least one oral glucose tolerance test (OGTT) drawn clinically within eight months of a fasting serum zinc assessment (See Table 1). Low serum zinc was observed in 10 out of 30 (33%) of the patients. Patients with low serum zinc had significantly lower fasting insulin, compared to those with normal serum zinc (*p* = 0.04, Table 1). Only three of the ten patients with abnormal fasting glucose values also had low serum zinc. Three subjects in whom OGTT results met the criteria for a diagnosis of diabetes, had serum zinc concentrations of 64.0, 68.5 and 74.0 μg/dL. 

**Table 1:** Demographics and Basic Laboratory Results for 30 Subjects with Transfusion Dependent Thalassemia from a Clinical Oral Glucose Tolerance Test.

Results	Total	Low Zinc	Normal Zinc	*p*-value
	*n* = 30	*n* = 10	*n* = 20	
Male, *n* (%)	16 (53%)	6 (60%)	10 (50%)	NS
Age, years	27.8 ± 9.1	29.2 ± 12.1	27.2 ± 7.4	NS
Serum Zinc, μg/dL	76.6 ± 10.5	67.7 ± 3.9	81.0 ± 9.9	0.003
Serum Copper, μg/dL	85.4 ± 25.4	86.6 ± 21.7	84.8 ± 27.6	NS
Height, cm	160.5 ± 9.4	162.9 ± 12.5	159.3 ± 7.5	NS
Weight, kg	57.8 ± 15.8	62.1 ± 22.6	55.7 ± 11.1	NS
Body Mass Index, kg/m^2^	22.3 ± 4.9	23.1 ± 7.0	21.9 ± 3.6	NS
Serum Ferritin, ng/mL *	2,752 ± 2,537	2,004 ± 1,713	3,146 ± 2,842	NS
Fasting Glucose, mg/dL	97.8 ± 15.1	99.0 ± 21.8	97.3 ± 11.1	NS
Abnormal Fasting Glucose, n (%)	11 (37%)	3 (30%)	8 (40%)	NS
Fasting Insulin, μIU/mL	6.2 ± 4.9	4.1 ± 2.6	7.2 ± 5.4	0.04
Glucose at 120 min, mg/dL	144 ± 57	165 ± 75	134 ± 45	0.16
Insulin at 120 min, μIU/mL	35.8 ± 50.1	30.6 ± 24.1	38.3 ± 50.4	NS
Insulin/Glucose at 120 min	0.239 ± 263	0.207 ± 0.152	0.255 ± 0.302	NS
Mean Glucose, mg/dL	136 ± 33	109 ± 44	97 ± 27	NS
Mean Insulin, μIU/mL	32 ± 29	21 ± 13	37 ± 39	NS
Glucose, Area Under the Curve	15,316 ± 5,062	14,768 ± 7,629	15,590 ± 3,363	NS
Insulin, Area Under the Curve	4,222 ± 4,055	2,965 ± 1,557	4,962 ± 4,877	0.13
Measures of Insulin Sensitivity				
HOMA-IR	1.49 ± 1.18	1.01 ± 0.67	1.74 ± 1.32	0.11
Insulin Sensitivity Index (ISI)	9.09 ± 4.90	11.38 ± 5.01	7.95 ± 4.54	0.06
Measure of Beta Cell Function				
Insulinogenic Index (IGI)	0.66 ± 0.65	0.32 ± 0.38	0.84 ± 0.70	0.036
Disposition Index (ODI)	4.44 ± 4.09	2.23 ± 2.73	5.50 ± 4.27	0.046

Mean ± SD, *p*-values determined from *t*-tests; *Serum Ferritin: Average of last three clinically drawn serum ferritin results from prior to OGTT; HOMA-IR: Homeostatic model assessment-insulin resistance; ODI: Oral Glucose Tolerance Test Disposition Index. Data from one subject in low zinc group with ODI 22.6 was excluded. This value was 3.8-fold greater than the next highest value in the group (5.99).

**Figure 1:**
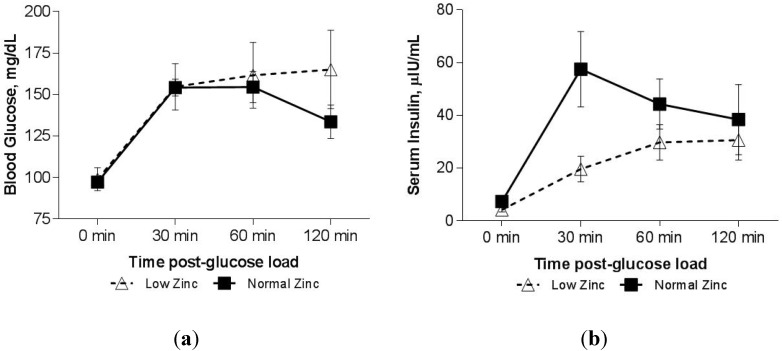
(**a**) Blood glucose response to a 75-g oral glucose load in patients with Thalassemia with normal (*n* = 20) or low serum zinc (*n* = 10). Using analysis of variance, a trend towards a Zinc × time effect was observed, *p* = 0.10. Abnormal blood glucose level at 120 min is considered any value >140 mg/dL. Data presented as Mean ± SEM. Using analysis of covariance adjusted for baseline, a significant Zinc × time effect was observed, *p* = 0.048. There is a significant peak of glucose at 30 and 60 min post 75 g dose in patients with Normal Zn, which comes back to near baseline at 120 min. However, with patients with Low Zn, there are no significant differences at any of the time points post dose. **(b**) Serum insulin response to a 75-g oral glucose load in transfused patients with Thalassemia who had normal (*n* = 20) or low serum zinc (*n* = 10). Insulin Values above are raw Means ± SEM; Using analysis of covariance, adjusted for baseline, a trend towards a significant Zinc × time effect was observed, *p* = 0.06.

**Figure 2:**
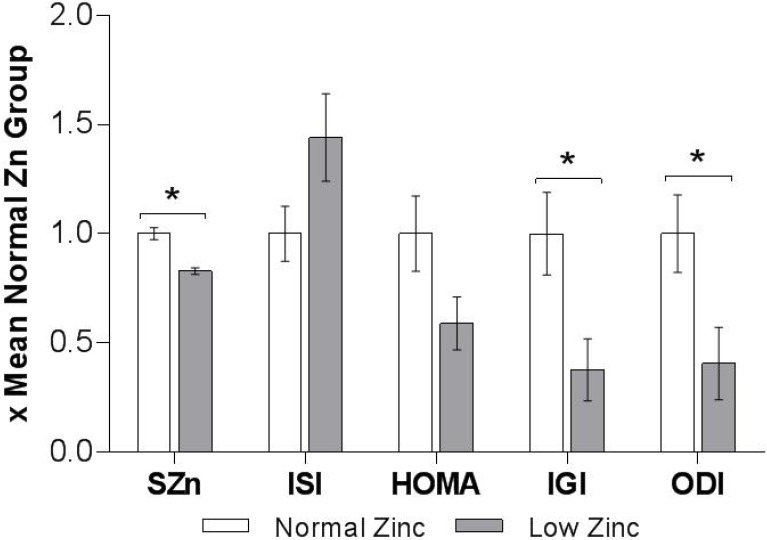
Oral glucose tolerance test derived indices of insulin sensitivity and beta xell function in normal (*n* = 20) and low zinc (*n* = 10) groups. All indices were normalized to mean of 1 for the normal zinc group and shown as mean ± SEM. * indicates statistically significant difference by unpaired *t*-test (*p* < 0.05). SZn: Serum Zn, ISI: insulin sensitivity index (Matsuda), HOMA: Homeostatic Model Assessment—Insulin Resistance, IGI: Insulinogenic index, ODI: Oral Disposition Index. One subject with ODI > 4.0 in low zinc group was excluded from the analysis (see footnote to Table 1).

We observed that the glucose response curve following OGTT in those with low serum zinc was altered. At the 2-h time-point, serum glucose had returned to near fasting level in those with normal serum zinc, but remained elevated in those with low serum zinc (group × time interaction *p* = 0.048, Figure 1a). Those with normal serum zinc concentration had a significant decrease in glucose concentration with time (after controlling for baseline), however those with low circulating zinc did not (Figure 1a), so that at 120 minutes, the mean (±SEM) glucose level in the low zinc group was higher (165.1 ± 23.6 mg/dL) compared with the normal zinc group (133.6 ± 10.1 mg/dL, *p* = 0.05). We observed a borderline effect of a lower insulin response to the glucose load in the low zinc group after controlling for baseline (Figure 1b; group × time interaction, *p* = 0.06), though none of the *post hoc* analyses to assess the time effect adjusting for multiple co compared with normal zinc group (57.5 ± 14.3 μIU/mL, *p* = 0.018). As the rise in blood glucose from 0–30 min was similar in both groups; the Insulinogenic Index (IGI, mean ± SEM) in the low zinc group (0.32 ± 0.12) was only 37% of the normal zinc group (0.84 ± 0.16, *p* = 0.036). There was no evidence of hepatic insulin resistance in either of the groups when measured as HOMA-IR (Table 1). In fact, the Matsuda whole body Insulin Sensitivity Index (ISI) was slightly higher in the low zinc group (11.4 ± 1.6 *versus* normal zinc group 8.0 ± 1.0, *p* = 0.06), while the Oral Disposition Index was reduced (2.23 ± 0.91 in low zinc group *versus* 5.50 ± 0.98, in normal zinc group, *p* = 0.046, Figure 2).mparisons proved significant. Using unpaired *t*-tests, the insulin response at 30 min was significantly smaller in the low zinc group (19.6 ± 4.9 IU/mL) 

**Figure 3:**
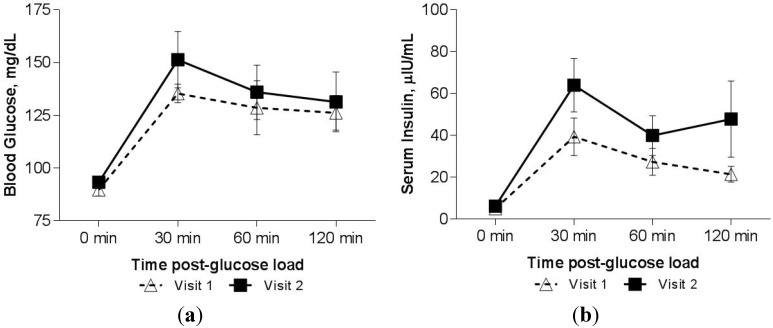
(**a**) Blood glucose response to a 75-g oral glucose load in *n* = 5 patients with Thalassemia who had a decline in serum zinc, measured at two separate visits, two years apart. Values above are mean blood glucose ± SEM. (**b**) Serum insulin response to a 75-g oral glucose load in *n* = 5 patients with Thalassemia who had a decline in serum zinc, measured at two separate visits, two years apart. Insulin values above are raw means ± SEM.

The effect of decline in serum zinc over time on OGTT was analyzed in five subjects (three female). Over an average period of two years, serum zinc decreased by 19.0 ± 9.6 μg/dL (*p* = 0.01, Table 2) and serum copper by 24.2 ± 18.7 μg/dL (*p* = 0.08). Chelation dosages were increased markedly during this time interval for one patient in response to rising iron load, while one other subject started growth hormone therapy. Overall, the effect of developing zinc deficiency on OGTT response measured by paired samples was different from the cross-sectional group. An increase in blood glucose manifesting as 7% increase in glucose AUC was observed in Visit 2 compared to Visit 1 (Figure 3a) while insulin AUC increased by 67% (Figure 3b). The pre-visit differed from the post-visit insulin response to the OGTT (after controlling for time post glucose load in a repeated measures ANOVA model, *p* = 0.035). ISI decreased from an average (±SD) of 9.9 ± 4.8 to 6.0 ± 1.6 (*p* = 0.11) with no change in HOMA-IR. Moreover, both the fasting blood glucose (*p* = 0.06) and the ratio of insulin/glucose at the final time point of the OGTT (120 min, *p* = 0.05) consistently increased in all patients as serum zinc dropped. 

**Table 2:** Clinical data in five patients with transfusion dependent Thalassemia with repeat oral glucose tolerance test and serum zinc concentrations.

ID	OGTT Test	Age, Year	Body Mass Index, kg/m^2^	Serum Zinc, μg/dL	Ferritin μg/L	Fasting Blood Glucose mg/dL	HOMA-IR	AUC Glucose mg/dL	Insulin/Glucose @120 min	ISI	ODI	IGI	Chelation, (mg/kg)
I	Pre	13	17.1	86.0	3,414	84.0	1.2	10,515	0.103	13.7	3.92	0.29	DFO (20)
	Post	17	19.2	75.0	7,920	86.0	0.9	13,830	0.233	8.0	3.46	0.43	DFO (31); DSX (39)
2	Pre	30	21.1	100.0	1,150	90.0	0.8	13,905	0.255	6.4	7.37	1.15	DFO (13); DSX (26)
	Post	32	20.4	67.0	1,100	91.0	1.2	18,420	0.661	4.7	5.31	1.13	DFO (9); DSX (32)
3	Pre	17	20.4	95.0	833	100.0	0.6	15,030	0.133	6.0	7.17	1.19	DFO (18); DSX (23)
	Post	18	18.5	71.0	1,180	101.0	0.5	11,115	0.230	7.3	53.5^$^	7.33	DFO (18); DFP (66)
4	Pre	25	23.7	71.0	278	84.0	1.9	15,705	0.162	14.0	5.23	0.37	DSX (7)
	Post	28	24.3	54.0	406	92.0	0.8	14,475	0.250	5.5	8.25	1.50	DSX (24)
5	Pre	16	20.3	73.0	1,090	91.0	0.6	13,020	0.189	7.0	5.38	0.77	DFO (12); DSX (32)
	Post	17	20.3	63.0	1,230	97.0	0.4	15,180	0.283	4.6	3.12	0.68	DSX (34)
Pre, Mean ± SD		20.2 ± 7.0	20.5 ± 2.4	85.0 ± 12.9	1,353 ± 1,202	89.8 ± 6.6	1.0 ± 0.5	13,635 ± 2,026	0.168 ± 0.058	9.4 ± 4.1	5.8 ± 1.4	0.8 ± 0.4	
Post, Mean ± SD		* 22.4 ± 7.1	20.5 ± 2.2	* 66.0 ± 8.1	2,367 ± 3,122	^ 93.4 ± 5.8	0.8 ± 0.3	14,604 ± 2,631	^ 0.331 ± 0.185	6.0± 1.6	5.0 ± 2.3	2.2 ± 2.9	

^ 9885st continues bces between erent from one another *p* < 0.05 by pairedntinuous variables, but dissimilar for two subjects. Where * Denotes Mean Pre/Post values different from one another (*p* < 0.05) by Students paired *t*-tests with two tailed distribution; ^ denotes a trend (*p* < 0.1); $: an outlier, not included in the summary statistics. Subject 3 was prescribed growth hormone between pre and post time points. Ferritin: average of three ferritin values prior to OGTT clinical exam date; AUC glucose: Area under the curve glucose. HOMA-IR: Homeostatic Model-Insulin Resistance [16]; IGI: Insulinogenic index, ODI: Oral Glucose Tolerance Test Disposition Index. ISI: insulin sensitivity index [16]; DFO: deferoxamine; DSX: deferasirox; DFP: deferiprone, Chelation dosages provided in the nearest integer.

## 4. Discussion

These preliminary results suggest that zinc status is related to glucose homeostasis and insulin secretion in patients with transfusion dependent thalassemia. It is clear that the glucose response curve differs between patients with low circulating zinc and those with normal zinc, a difference that has not been reported previously. For patients with low zinc, glucose remains elevated 2 h after a 75 g oral dose, while it returns to near baseline levels for those with normal zinc status. Moreover, the Insulinogenic and Disposition Indexes were both decreased in the low zinc group suggesting reduced beta cell function and impaired peripheral utilization of glucose. A previous report has suggested that patients with Thal and low zinc status have a stunted insulin response (secretion) to a glucose load [12], but glucose response was not reported. We observed a similar effect on insulin in this study (Figure 1b); however, our findings did not reach statistical significance, possibly because these subjects were only marginally zinc deficient, or due to our limited sample size. The observed results are consistent with the robust literature documenting the diabetogenic effects of altered zinc status on reduced insulin secretion and altered glucose disposal.

Perhaps the most unique evidence to support the link between zinc status and glucose homeostasis in this population was observed in the five subjects with repeat OGTT examinations. In these patients, zinc status dramatically declined over time, while fasting blood glucose and insulin/glucose ratios dependably increased over the same period. There was a trend towards an increased mean glucose concentration with elevated insulin response. These data may suggest the progressive nature by which diabetes develops in patients with Thal. Those with chronic, low zinc status (represented by the cross-sectional group, Table 1) may have dampened insulin secretion (Figure 1b), while those with recent marginal zinc depletion (paired longitudinal observations, Table 2) were still able to elicit a robust insulin response to the glucose load (Figure 3b). Decreased uptake of glucose in the periphery seems to be a common feature in both groups, and may be the underlying mechanism of the contribution of zinc status to pathogensis of diabetes. The need for greater insulin mediated by zinc deficiency could be a driver towards eventual beta cell dysfunction. 

The literature is rich with illustrations of the direct role zinc has in the development of or possible treatment for diabetes [9,10]. Insulin activity and release from the pancreas is reduced in zinc deficiency, but insulin synthesis is normal [18]. Zinc appears to be involved in the reduction or delay in the onset of diabetes by decreasing TNFα and IL-1, cytokines known for their role in beta-cell destruction [9]. Additionally, zinc may be involved not only in the protection of islet cells, but it is also crucial to the many steps of glucose metabolism and insulin signaling. At the most basic level, zinc binds to the insulin-receptor on the plasma membrane activating and thereby enabling the translocation of insulin into the cells [19]. Additionally zinc activates the insulin-response aminopeptidase (IRAP) molecule, which enables the translocation of GLUT4 to the cell surface for glucose transport into the cell [20]. As a result, supplementation with zinc has been shown to decrease hyperglycemia in existing Type II diabetes [10,21,22]. 

Elevated urinary excretion of zinc is a consistent finding in diabetic subjects [23]. Increased urinary zinc has been reported in patients with diabetes and thalassemia [24]. Increased liver zinc has been observed in diabetic mice [25], possibly due to an increase in metallothionein, but to date this has not been investigated in patients with Thal. It is quite possible this phenomenon occurs in heavily iron overloaded subjects with Thal, leading to a functional zinc deficiency though the concentration of zinc in the liver, or the transport of zinc out of tissues important for optimal zinc function, has never been systematically investigated. 

Marginal zinc status is extremely common in patients with Thal. Roughly 30% of patients in our clinic have low circulating zinc concentrations, despite the majority being prescribed a daily multi-vitamin mineral supplement that contains an additional 15 mg of zinc per day [11]. Since dietary and/or supplemental intake of zinc does not appear to be lacking for most patients [11,26], alternative etiologies must explain the high prevalence of this functional deficiency. For instance, increased utilization of zinc due to oxidative stress, increased urinary zinc excretion and sequestration in the liver related to increasing iron overload, may account for the zinc deficiency in Thal. 

The interpretation of OGTT can be challenging in patients with Thal given they may exhibit a combination of decreased insulin secretion and impaired peripheral utilization of glucose in the face of elevated oxidative stress. In this study, some of the typical clinical tools, fasting glucose and insulin, HOMA-IR, and ISI were not consistently predictive for the assessment of beta cell and pancreatic function. Therefore, relying on clinical information from fasting samples from patients provides limited data. The insulin to glucose response after a glucose load (Insulinogenic Index) appears to be an early marker of beta cell dysfunction in Thal where fasting glucose is typically normal. Therefore, OGTT remains a critical end-point for patients with Thal to provide much needed clinical information regarding beta cell function, insulin sensitivity and glucose homeostasis. Diabetes in Thal is reversible if diagnosed early and whole body iron load reduced, but our data suggest that improvement in zinc status should also be intensely pursued to prevent this serious complication.

Iron overload is a known risk factor for diabetes in patients with Thal. In adults without Thal, elevated serum ferritin levels have been associated with impaired beta-cell function and decreased insulin sensitivity [27]. Given this, aggressive chelation therapy has been shown to reduce total body iron stores, and thereby decrease the risk of diabetes in patients with transfusion dependent Thal [28]. However, there is a balance, and intensive chelation therapy, especially in patients with low total body iron stores can lead to depletion of other essential trace minerals including zinc. Provided what is known about zinc and diabetes, clinicians should be sensitive to the zinc status of any patient with Thal on combination or intensive chelation therapy.

Although this study is preliminary and limited by the retrospective design and small sample size, the dramatic association of concurrent decline of circulating zinc in a subgroup of patients with altered glucose homeostasis is quite provocative. The paired results are complicated somewhat by one subject who initiated growth hormone therapy between the two visits, as growth hormone is well known to effect glucose disposal [29].

In conclusion, patients with transfusion dependent thalassemia who were marginally zinc deficient had lower secretion of insulin and an impaired glucose response curve following an oral glucose challenge than those with normal zinc. Future investigations of zinc metabolism will require modeling of OGTT data and not simply measurement of static indices in order to understand the complexities of pancreatic function in the Thal patient.
